# Dynamic Effective Connectivity using Physiologically informed Dynamic Causal Model with Recurrent Units: A functional Magnetic Resonance Imaging simulation study

**DOI:** 10.3389/fnhum.2023.1001848

**Published:** 2023-03-01

**Authors:** Sayan Nag, Kamil Uludag

**Affiliations:** ^1^Techna Institute & Koerner Scientist in MR Imaging, University Health Network, Toronto, ON, Canada; ^2^Department of Medical Biophysics, University of Toronto, Toronto, ON, Canada; ^3^Center for Neuroscience Imaging Research, Institute for Basic Science, Suwon, Republic of Korea; ^4^Department of Biomedical Engineering, Sungkyunkwan University, Suwon, Republic of Korea

**Keywords:** dynamic effective connectivity, neuroscience, graphical models, BOLD fMRI, causality

## Abstract

Functional MRI (fMRI) is an indirect reflection of neuronal activity. Using generative biophysical model of fMRI data such as Dynamic Causal Model (DCM), the underlying neuronal activities of different brain areas and their causal interactions (i.e., effective connectivity) can be calculated. Most DCM studies typically consider the effective connectivity to be static for a cognitive task within an experimental run. However, changes in experimental conditions during complex tasks such as movie-watching might result in temporal variations in the connectivity strengths. In this fMRI simulation study, we leverage state-of-the-art Physiologically informed DCM (P-DCM) along with a recurrent window approach and discretization of the equations to infer the underlying neuronal dynamics and concurrently the dynamic (time-varying) effective connectivities between various brain regions for task-based fMRI. Results from simulation studies on 3- and 10-region models showed that functional magnetic resonance imaging (fMRI) blood oxygenation level-dependent (BOLD) responses and effective connectivity time-courses can be accurately predicted and distinguished from faulty graphical connectivity models representing cognitive hypotheses. In summary, we propose and validate a novel approach to determine dynamic effective connectivity between brain areas during complex cognitive tasks by combining P-DCM with recurrent units.

## 1. Introduction

Functional Magnetic Resonance Imaging (fMRI) non-invasively measures neural activity indirectly *via* changes in the hemodynamic response (i.e., changes in cerebral blood flow and volume). Local blood brain flow increases when the neuron increases its activity in the presence of a stimulus or intrinsically to support the increased metabolic demand and subsequently oxygenated blood displaces deoxygenated blood (Buxton et al., 2004; Stefanovic et al., 2004; Uludağ et al., 2004). This leads to a rise in the blood oxygenation level-dependent (BOLD) response during stimulation before the response typically falls to a post-stimulus undershoot below the initial baseline and ultimately returns to baseline. The BOLD signal is not only used to map task-correlated brain activity or study brain physiology in individual voxels but also used to study functional and effective connectivity (Friston, 2011; Goldenberg and Galván, 2015; Kuhnke et al., 2021; Underwood et al., 2021). Functional connectivity is another term for instantaneous BOLD signal correlations during resting-state of remote voxels and brain areas (van den Heuvel and Pol, 2010; Friston, 2011; Shakil et al., 2016). However, functional connectivity methods typically are not utilized to infer causal relationships between these voxels (Friston, 2011). In contrast, effective connectivity utilizes causal connectivity graphs on the neuronal level representing a specific cognitive hypothesis for a task and a local generative model numerically describing the underlying BOLD signal physiology (Friston, 2011).

One prominent approach for effective connectivity is the Dynamic Causal Model (DCM) (Friston et al., 2003; Stephan et al., 2008; Moran et al., 2009; Havlicek et al., 2015). The basic idea behind DCM is to treat the brain as a nonlinear dynamical system and the observations (e.g., whole-brain fMRI signals) as indirect reflections of the signal of interest (e.g., the local neuronal activity and their connections). Using Bayesian model inversion (Ulrych et al., 2001; Friston et al., 2003), the local neuronal, effective connectivity values, and the vascular parameters can be estimated (Friston et al., 2003; Havlicek et al., 2015). The variants of DCM include stochastic DCM (Daunizeau et al., 2009), non-linear DCM (Stephan et al., 2008), spectral DCM (Moran et al., 2009) and physiologically informed DCM (P-DCM) (Havlicek et al., 2015). The P-DCM is the state-of-the-art model, which is inspired by experimental observations about the physiological underpinnings of the fMRI signal. It overcomes the limitations of earlier DCMs, such as inaccurate modeling of the initial overshoot and the post-stimulus undershoot, observations which are typically present in the time courses of task-based fMRI BOLD signals.

Dynamic Causal Model studies typically consider the causal interactions between brain areas to be fixed for an entire experimental run. However, experimental conditions can change with time within a run, for example when a subject is watching a movie, and consequently it is expected that the connectivity strengths between disparate brain regions also vary with time. In the recent years, more and more studies utilize these time-varying stimuli to study cognitive processes in the human brain (see, for example, Finn, 2021). To capture the dynamic nature of functional connectivity in resting-state, Dynamic Functional Connectivity (DFC) studies using sliding windows were proposed (Chang and Glover, 2010; Kiviniemi et al., 2011; Jones et al., 2012; Shakil et al., 2016). This is done by finding the statistical correlations amongst different brain area-specific resting-state fMRI BOLD time-series (Cribben et al., 2012; Handwerker et al., 2012; Calhoun et al., 2014; Monti et al., 2014; Shakil et al., 2016). However, as this analysis is done on the level of observations and, hence, does not utilize a generative model of the BOLD signal, DFC does not provide an assessment of the underlying neuronal mechanisms reflected in the fMRI BOLD responses (Stephan et al., 2010; Friston, 2011).

In this study, we propose a P-DCM based Dynamic Effective Connectivity approach the for modeling underlying neuronal dynamics in task-based fMRI. Our approach consists of sliding (recurrent) overlapping windows to capture the entire extent of fMRI BOLD time-series in a sequential manner. For each window, we have used discretized P-DCM with different parameter sets (i.e., connectivity variables, neuronal and vascular parameters) for different windows following a recurrence (from the previous window). Finally, for each such recurrent unit, we perform model inversion (parameter estimation) until convergence.

## 2. Methods

To determine time varying connectivity, we combined two approaches: discretized Physiologically informed Dynamic Causal Model (*d*P-DCM) and Recurrent Unit (RU).

### 2.1. *discretized* Physiologically informed Dynamic Causal Model (*d*P-DCM)

Physiologically informed Dynamic Causal Model (P-DCM) for fMRI was introduced by Havlicek et al. (2015) to describe the link between hidden neuronal activity and measured BOLD signals, overcoming the physiological limitations of previous Dynamic Causal Models (DCMs). The drawbacks of previous models included inaccurate modeling of initial overshoot and post-stimulus undershoot, which are temporal features typically observed in task-based fMRI BOLD signals.

The DCM approaches consist of a forward generative model and a backward model or inference (Friston et al., 2003; Havlicek et al., 2015). The P-DCM forward model (see Havlicek et al., 2015) consists of a two-state excitatory-inhibitory neuronal model which incorporates adaptive neuronal dynamics, capable of reproducing local field potential time courses as observed with invasive electrophysiology; neurovascular coupling is described as a feed-forward mechanism, and cerebral blood flow and volume can be uncoupled; and finally, Havlicek et al. also derived new parameters for the BOLD signal equation. Importantly, it has been shown that the different physiological assumptions of P-DCM compared with previous DCM approaches can lead to different estimated effective connectivity values (please see Havlicek et al., 2017b, for details).

To utilize RUs, we used a *discretized* version of P-DCM. For simplicity, instead of locally linearize the matrix exponential (Ozaki, 1992), we have used the Euler’s method.


(1) $$\frac{d\,z\,\left(t\right)}{d\,t}=\frac{z\,\left(t+\Delta \,t\right)-z\,\left(t\right)}{\Delta \,t}$$

Where *z* ∈ {*x*_*E*_, *x*_*I*_, *a*, *f*, *v*, *q*}, *x*_*E*_ the excitatory and *x*_*I*_ the inhibitory neuronal response, *a* the vasoactive signal, *f* the normalized cerebral blood flow response, *v* the normalized cerebral blood volume response, *q* the normalized deoxyhemoglobin content and Δ*t* the difference between two adjacent time-points.

#### 2.1.1. Neuronal component

The neuronal component estimates excitatory and inhibitory neuronal dynamics by modeling intra-regional and inter-regional neuronal interactions. The discretized neuronal component consisting of both excitatory (*x*_*E*_) and inhibitory neuronal states (*x*_*I*_) is given as (here and below, please see Havlicek et al., 2015, for the corresponding continuous variable equations):


(2a) $$x_{E}\;\left(t+\Delta \,t\right)=\left(1-\sigma \,\Delta \,t\right)\cdot x_{E}\,\left(t\right)-\left(\mu \,\Delta \,t\right)\cdot x_{I}\,\left(t\right)+\left(c\,\Delta \,t\right)\cdot u\,\left(t\right)$$

(2b) $$x_{I}\;\left(t+\Delta \,t\right)=\left(\lambda \,\Delta \,t\right)\cdot x_{E}\,\left(t\right)+\left(1-\lambda \,\Delta \,t\right)\cdot x_{I}\,\left(t\right)$$

Equation 2a refers to the excitatory neuronal dynamics and 2b refers to the inhibitory neuronal dynamics. σ denotes the self-connectivity whose magnitude determines the temporal scaling of the neuronal dynamics. λ is the inhibitory gain factor that controls relative amplitude of the inhibitory activity with respect to the excitatory activity and the temporal smoothness. μ represents the inhibitory-excitatory connection, which regulates the temporary imbalance of the excitatory activity due to the inhibition. λ and μ are also deciding the rate, at which the activity of excitatory neuronal response drops from its initial peak to the plateau level and its return from the post-stimulus dip to the baseline value.

A discretized version of the multivariate form of the two-state connectivity equation (Havlicek et al., 2015) based on the neuronal component (described in the equations 2a and 2b) is given as:


(2c) $${\text{X}}_{E}\;\left(t+\Delta \,t\right)=\left(\text{I}+\Delta \,t\,\psi \right)\,{\text{X}}_{E}\,\left(t\right)+\left(\Delta \,t\,\psi^{-}\right)\,{\text{X}}_{I}\,\left(t\right)+\left(\Delta \,t\,\text{C}\right)\,{\text{U}}_{d}\,\left(t\right)$$

(2d) $${\text{X}}_{I}\;\left(t+\Delta \,t\right)=\left(\Delta \,t\,ℭ\right)\,{\text{X}}_{E}\,\left(t\right)+\left(\text{I}-\Delta \,t\,ℭ\right)\,{\text{X}}_{I}\,\left(t\right)$$

for which


$$\begin{array}{c} \psi_{i\,j}={\text{A}}_{i\,j}+\sum_{m=1}^{M}{\text{B}}_{i\,j}^{\left(m\right)}\,u_{m}\,\left(t\right),\\ \psi_{i\,j}^{-}=0,\\ {ℭ}_{i\,j}=0, \end{array}\}\,\forall i\neq j,$$

$$\begin{array}{c} \psi_{i\,i}=-\sigma \,e^{\left(\tilde{\sigma }+\sum_{m=1}^{M}b_{i\,i}^{\left(m\right)}\,u_{m}\,\left(t\right)\right)},\\ \psi_{i\,i}^{-}=-\mu \,e^{\left(\tilde{\mu_{i}}+\sum_{k=1}^{K}b_{\mu \,i}^{\left(k\right)}\,u_{\mu \,k}\,\left(t\right)\right)},\\ {ℭ}_{i\,i}=\lambda \,e^{\left(\tilde{\lambda_{i}}+\sum_{l=1}^{L}b_{\lambda \,i}^{\left(l\right)}\,u_{\lambda \,l}\,\left(t\right)\right)} \end{array}\}$$

**A** is the connectivity matrix [whose off-diagonal elements encode connections between regions whereas diagonal elements encode self-connections (Havlicek et al., 2015)], **B** denotes the matrix consisting of the additive modulatory effects controlled by modulatory inputs *u*_*m*_(*t*), and ψ is the total connectivity matrix. The direct input stimulus matrix is given as **U***_d_*(*t*). The context dependent inputs are represented as *u_μ__*k*_*(*t*) and *u_λ_*_*l*_(*t*), which are scaled by region-specific parameters *b_μ__*i*_*^(^*^k^*^)^ and *b_λ_*_*i*_^(^*^l^*^)^, and together they modulate the inhibitory-excitatory connections and inhibitory gain factors, respectively. These factors are encoded in the matrix given by ℭ. **I** is the identity matrix. In the above equations (derived from Havlicek et al., 2015), the parameters $\tilde{\sigma ,}\,\tilde{\mu ,}\,\tilde{\lambda }$ represent self-connectivity, inhibitory-excitatory connection, and inhibitory gain, respectively, and σ,μ,λ are the corresponding constant scaling factors (please refer to Havlicek et al., 2015 for further details). Equations 2c and 2d can be represented in matrix form as follows:


(2e) [XE⁢(t+Δ⁢t)XI⁢(t+Δ⁢t)]=[I+△⁢t⁢ψΔ⁢t⁢ψ-Δ⁢t⁢ℭI-Δ⁢t⁢ℭ]⁢[XE⁢(t)XI⁢(t)]+[Δ⁢t⁢C0]⁢Ud⁢(t)

(2f) $$\text{X}\,\left(t+\Delta \,t\right)={\text{W}}_{X}\,\text{X}\,\left(t\right)+{\text{W}}_{U}\,{\text{U}}_{d}\,\left(t\right)$$

In the equation 2f, **X** represents the excitatory and inhibitory neuronal variables stacked together in a matrix form, **W***_X_* and **W***_U_* represent the collective matrices of individual weight matrices of **X**(*t*) and **U***_d_*(*t*), respectively.

#### 2.1.2. Feedforward neurovascular coupling (NVC) component

Neurovascular coupling is the relationship between local neuronal activity and subsequent changes in CBF occurring through a complex sequence of coordinated events involving neurons, glia, and vascular cells. That is, neuronal excitation/inhibition leads to arterial vasodilation/vasoconstriction associated with increased/decreased CBF (Zonta et al., 2003; Uludağ et al., 2004; Lauritzen, 2005; Devor et al., 2007; Attwell et al., 2010)—with the result that the CBF time course is a smoothed version of the neuronal activity. Considering the constraint of linear relationship between synaptic activity and blood flow, the discretized version of feedforward NVC component can be given as:


(3a) $$a\,\left(t+\Delta \,t\right)=\left(1-\varphi \,\Delta \,t\right)\cdot a\,\left(t\right)+\left(\Delta \,t\right)\cdot x_{E}\,\left(t\right)$$

(3b) f⁢(t+Δ⁢t)=(ϕ⁢Δ⁢t)⋅a⁢(t)+(1-χ⁢Δ⁢t)⋅f⁢(t)+χ⁢Δ⁢t

Here, *a*(*t*) is the time-varying vasoactive signal responsible for transforming the excitatory neuronal response *x*_*E*_(*t*) to the CBF response *f*(*t*). The set of equations 3a and 3b acts as a positively constrained low-pass filter of the neuronal dynamics as regulated by vasoactive signal decay (φ), vasoactive signal gain (ϕ) and blood inflow signal decay (*χ*).

The above set of equations in matrix form can be written as:


(3c) [a⁢(t+Δ⁢t)f⁢(t+Δ⁢t)]=[ 1-φ⁢Δ⁢t       0       ϕ⁢Δ⁢t    1-χ⁢Δ⁢t]⁢[a⁢(t)f⁢(t)]+[Δ⁢t0] xE⁢ (t) +[0χ⁢Δ⁢t]

#### 2.1.3. Hemodynamic component

The CBF response *f*(*t*) acts as an input to the post-capillary vessels, which are represented by an expandable venous balloon. The system of equations governing the hemodynamics describes the interaction between blood inflow *f*(*t*), blood outflow *f*_*out*_(*t*), blood volume *v*(*t*) and deoxyhemoglobin content *q*(*t*) as they flow through the venous balloon. The discretized version of the set of equations is given as:


(4a) $$v\;\left(t+\Delta \,t\right)=v\,\;(t)+\Delta \,t\cdot \left[\frac{f\,\;(t)-f_{o\,u\,t}\,\left(v,t\right)}{t_{M\,T\,T}}\right]$$

(4b) $$q\,\;(t+\Delta \,t)=q\,\;(t)\cdot \left[1-\frac{\Delta \,t\cdot f_{o\,u\,t}\,\left(v,t\right)}{t_{M\,T\,T,\cdot }\,v\,\left(t\right)}\right]+\Delta \,t\cdot \frac{f\,\;(t)\cdot E\,\left(f\right)}{t_{M\,T\,T}\cdot E_{0}}$$

(4c) $$E\;\left(f\right)=1-{\left(1-E_{0}\right)}^{1/f}$$

These equations are following mass balance principles: The blood volume *v*(*t*) depends on the difference between the blood inflow *f*(*t*) and the blood outflow *f*_*out*_(*t*). The deoxyhemoglobin content *q*(*t*) depends on the difference between the delivery rate of deoxyhemoglobin into the venous compartment and the rate of clearance of deoxyhemoglobin from the tissue. The scaling factor *t*_*MTT*_ denotes the mean transit time that blood takes to pass through the veins. *E*(*f*) represents the oxygen extraction fraction and *E*_0_ is the net oxygen extraction at rest. [Please note that it is easy to use a different relationship between *E* and *f*. However, for consistency with previous papers (Havlicek et al., 2015), we employ equation (4c) for this relationship]. In addition to this steady-state relationship, some studies indicate a tight temporal association (but not necessarily mechanistic coupling) between CBF and cerebral metabolic rate of oxygen (CMRO_2_) (Buxton and Frank, 1997; Masamoto et al., 2008; Zappe et al., 2008; Havlicek et al., 2017a).

#### 2.1.4. Balloon model with viscoelastic effect

Instead of using the steady state flow-volume relationship as used in the earlier versions of DCMs as in Friston et al. (2003), Stephan et al. (2008), Havlicek et al. (2015) considered the original balloon model with viscoelastic effect. It was experimentally revealed in Mandeville et al. (1998) that the steady-state power law relationship does not adequately describe the temporal properties of the CBF-CBV relationship (see Uludağ and Blinder, 2018, for a recent review). The post-stimulus BOLD undershoot, for example, is primarily due to slow recovery of venous CBV to baseline rather than a metabolic effect [(Buxton, 2012), but see van Zijl et al. (2012) for opposing view]. Considering the dynamic viscoelastic effect term leads to:


$$f_{o\,u\,t}\;\left(v,t\right)=v\;{\left(t\right)}^{\frac{1}{\alpha }}+\tau \cdot \frac{d\,v\;\left(t\right)}{d\,t}$$

(5a) $$=\frac{1}{\tau +t_{M\,T\,T}}\cdot \left(t_{M\,T\,T}\cdot v\,{\left(t\right)}^{\frac{1}{\alpha }}+\tau \cdot f\,\left(t\right)\right)$$

Here, α is Grubb’s exponent, which describes the stiffness of the vessel. The value of α was experimentally found to be about 0.38 (Grubb et al., 1974; Chen and Pike, 2009) but lower values have also been found, especially for short stimuli (see Uludağ and Blinder, 2018, for an overview). τ indicates the viscoelastic time constant, which controls the duration of transient adjustment of the shape of the venous balloon. The value of the viscoelastic time constant τ is non-zero and thus cerebral blood outflow follows a different curve than the inflow, resulting in a temporal uncoupling of CBF and CBV.

Combining equations 4a, 4b, 4c, and 5 in matrix form we get,


[v⁢(t+Δ⁢t)q⁢(t+Δ⁢t)]=

$$\left[\begin{array}{ccccccc} 1 & 0 & \left(\frac{△\,t}{t_{M\,T\,T}}-\frac{\tau \,△\,t}{\tau +t_{M\,T\,T}}\right) & -\frac{t_{M\,T\,T}\,△\,t}{\tau +t_{M\,T\,T}} & 0 & 0 & 0\\ 0 & 1 & 0 & 0 & \frac{t_{M\,T\,T}\,△\,t}{t_{M\,T\,T}\,\left(\tau +t_{M\,T\,T}\right)} & \frac{\tau \,△\,t}{t_{M\,T\,T}\,\left(\tau +t_{M\,T\,T}\right)} & \frac{△\,t}{t_{M\,T\,T}} \end{array}\right]$$

(5b) $$\left[\begin{array}{c} v\,\left(t\right)\\ q\,\left(t\right)\\ f\,\left(t\right)\\ v\,{\left(t\right)}^{\frac{1}{\alpha }}\\ \frac{q\,\left(t\right)\,v\,{\left(t\right)}^{\frac{1}{\alpha }}}{v\,\left(t\right)}\\ \frac{q\,\left(t\right)\,f\,\left(t\right)}{v\,\left(t\right)}\\ \left(\frac{1-{\left(1-E_{0}\right)}^{\frac{1}{f\,\left(t\right)}}}{E_{0}}\right)\,f\,\left(t\right) \end{array}\right]\;$$

Combining equations 3c and 5b we get,


$$\left[\begin{array}{c} a\,\left(t+\Delta \,t\right)\\ f\,\left(t+\Delta \,t\right)\\ v\,\left(t+\Delta \,t\right)\\ q\,\left(t+\Delta \,t\right) \end{array}\right]=$$

$$[\begin{array}{cccccc} 1-\varphi \,\Delta \,t & 0 & 0 & 0 & 0 & 0\\ \phi \,\Delta \,t & 1-\chi \,\Delta \,t & 0 & 0 & 0 & 0\\ 0 & \left(\frac{△\,t}{t_{M\,T\,T}}-\frac{\tau \,△\,t}{\tau +t_{M\,T\,T}}\right) & 1 & 0 & -\frac{t_{M\,T\,T}\,△\,t}{\tau +t_{M\,T\,T}} & 0\\ 0 & 0 & 0 & 1 & 0 & \frac{t_{M\,T\,T}\,△\,t}{t_{M\,T\,T}\,\left(\tau +t_{M\,T\,T}\right)} \end{array}\begin{array}{cc} 0 & 0\\ 0 & 0\\ 0 & 0\\ \frac{\tau \,△\,t}{t_{M\,T\,T}\,\left(\tau +t_{M\,T\,T}\right)} & \frac{△\,t}{t_{M\,T\,T}} \end{array}]$$

(5c) $$\left[\begin{array}{c} a\;\left(t\right)\\ f\,\left(t\right)\\ v\,\left(t\right)\\ q\,\left(t\right)\\ v\,{\left(t\right)}^{\frac{1}{\alpha }}\\ \frac{q\,\left(t\right)\,v\,{\left(t\right)}^{\frac{1}{\alpha }}}{v\,\left(t\right)}\\ \frac{q\,\left(t\right)\,f\,\left(t\right)}{v\,\left(t\right)}\\ \left(\frac{1-{\left(1-E_{0}\right)}^{\frac{1}{f\,\left(t\right)}}}{E_{0}}\right)\,f\,\left(t\right) \end{array}\right]+\left[\begin{array}{c} \begin{array}{c} \Delta \,t\\ 0\\ 0 \end{array}\\ 0 \end{array}\right]\;x_{E}\;\left(t\right)+\;\left[\begin{array}{c} \begin{array}{c} 0\\ \chi \,\Delta \,t\\ 0 \end{array}\\ 0 \end{array}\right]$$

The above equation 5c can be further simplified in matrix form to:


(5d) $$\text{H }\left(t+\Delta t\right)=\;{\text{W}}_{H}\theta_{H}\;(\text{H}\left(t\right))+{\text{W}}_{H\,X}x_{E}\;(t)+\omega_{H}$$

for which


(5e) $$\text{H }\left(t\right)=\left[\begin{array}{c} a\;\left(t\right)\\ f\,\left(t\right)\\ v\;\left(t\right)\\ q\,\left(t\right) \end{array}\right]$$

and


(5f) $$\theta_{H}\;(\text{H}\;(t))=[\begin{array}{cccccc} a\;\left(t\right)\;f\;\left(t\right)\;v\;\left(t\right)\;q\;\left(t\right)\;v\;{\left(t\right)}^{\frac{1}{\alpha }}\,\frac{q\,\left(t\right)\,v\,{\left(t\right)}^{\frac{1}{\alpha }}}{v\,\left(t\right)} & & & & & \end{array}$$

$$\begin{array}{cc} \frac{q\,\left(t\right)\,f\,\left(t\right)}{v\,\left(t\right)}\,\left(\frac{1-{\left(1-E_{0}\right)}^{\frac{1}{f\,\left(t\right)}}}{E_{0}}\right)\,f\;\left(t\right) & \end{array}]{}^{T}$$

**W***_H_* is weight matrix of the neurovascular coupling, hemodynamic and Balloon model parameters collectively given by **H** (Equation 5e), **W***_HX_* is the column matrix connecting the excitatory neuronal response *x*_*E*_ with **H** and *ω_*H*_* is the constant term. θ*_H_*(**H**(*t*)) is the column matrix consisting of the combination of the variables as shown in the equation 5f.

#### 2.1.5. BOLD signal component

The BOLD signal *y(t)* is determined by *v*(*t*) and *q*(*t*).


(6a) $$y\;\left(t\right)=V_{0}\,\left[k_{1}\cdot \left(1-q\,\left(t\right)\right)+k_{2}\cdot \left(1-\frac{q\,\left(t\right)}{v\,\left(t\right)}\right)+k_{3}\cdot \left(1-v\,\left(t\right)\right)\right]$$

(6b) $$k_{1}=4.3\cdot \vartheta_{0}\cdot E_{0}\cdot T\,E,$$

$$k_{2}=\varepsilon \cdot r_{0}\cdot E_{0}\cdot T\,E,$$

$$k_{3}=1-\varepsilon$$

Here, *V*_0_ is the resting venous blood volume fraction and *k*_1_, *k*_2_, *k*_3_ are dimensionless constants, which are dependent on the physiological properties of brain tissue and acquisition parameters of the Gradient Echo (GE) sequence. ε refers to the ratio of intra- to extravascular fMRI signal contributions. *ϑ_0_* symbolizes the field-dependent frequency offset at the outer surface of the magnetized blood vessel for fully deoxygenated blood. *r*_0_ is the regression slope of the relation between the variations in intravascular signal relaxation rate and alterations in oxygen saturation. *TE* denotes the echo time (in ms). The first term in the equation 6a describes the relationship of the extravascular signal and the deoxyhemoglobin content, the second term of the intravascular signal and the ratio between deoxyhemoglobin content and venous blood volume, and the third term depicts the volume-weighted balance between extravascular and intravascular signals. The values of the parameters in Equation (6b) for various field strengths can be found in Havlicek et al. (2015).

The above equation 6a can be written in the following matrix form:


(6c) $$y\;\left(t\right)=\left[\begin{array}{ccc} -V_{0}\,k_{1} & -V_{0}\,k_{2} & -V_{0}\,k_{3} \end{array}\right]\,\left[\begin{array}{c} q\;\left(t\right)\\ \frac{q\,\left(t\right)}{v\,\left(t\right)}\\ v\;\left(t\right) \end{array}\right]+V_{0}\,\left(k_{1}+k_{2}+k_{3}\right)$$

(6d) $$y\left(t\right)=\;{\text{W}}_{B\,O\,L\,D}\theta_{B\,O\,L\,D}\left(\left[\begin{array}{c} q\,\left(t\right)\\ v\,\left(t\right) \end{array}\right]\right)+\;\omega_{B\,O\,L\,D}$$

In total, the discretized P-DCM (*d*P-DCM) can be represented using the following set of equations:


(7) $$d\,P-D\,C\,M=\left\{ \begin{array}{c} \text{X}\left(t+\Delta t\right)=\;{\text{W}}_{X}\text{X}\left(t\right)+{\text{W}}_{U}{\text{U}}_{d}\left(t\right)\\ \text{H }\left(t+\Delta t\right)=\;{\text{W}}_{H}\theta_{H}\left(\text{H }\left(t\right)\right)+{\text{W}}_{H\,X}x_{E}\;(t)+\omega_{H}\\ y\;(t+\Delta t)=\;{\text{W}}_{B\,O\,L\,D}\theta_{B\,O\,L\,D}\left(\left[\begin{array}{c} q\,\left(t+\Delta \,t\right)\\ v\,\left(t+\Delta \,t\right) \end{array}\right]\right)+\;\omega_{B\,O\,L\,D} \end{array}\right.$$

### 2.2. Time varying calculations of the model parameters

**FIGURE 1:**
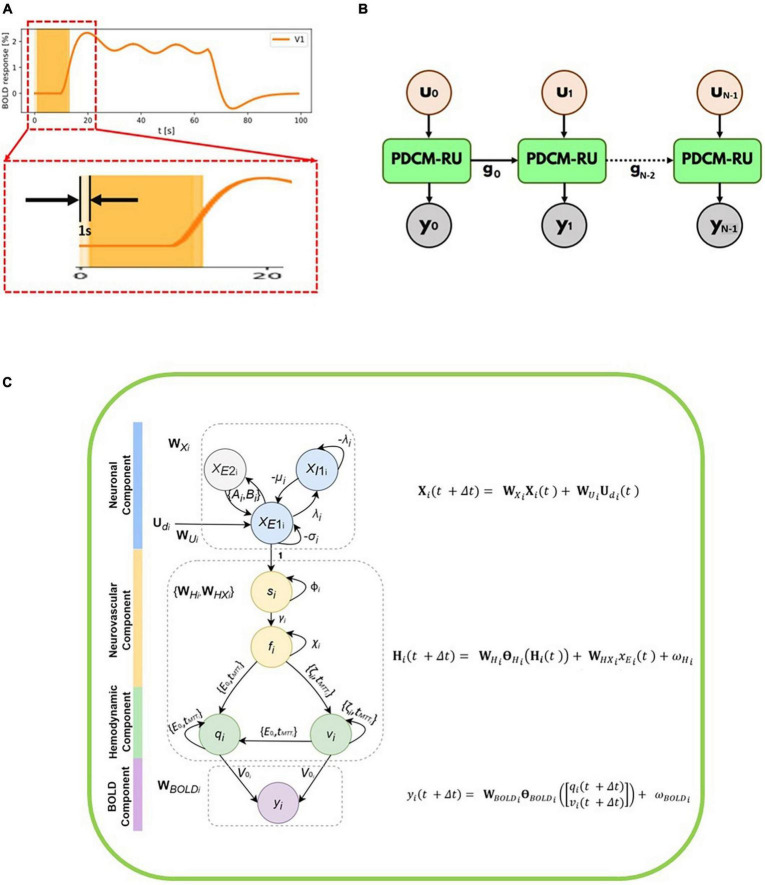
**(A)** Simulated BOLD fMRI time-courses for R1 along with two overlapping windows each of 12 s duration which moves from left to right. The time course between 0 and 20 seconds has been magnified to show the overlap of two successive windows. The stride of each overlapping window is 1 s. **(B)** Unfolded version of the *discretized* Physiologically informed Dynamic Causal Model (*d*PDCM)-recurrent unit (RU) architecture through time. For *i*^th^ window for which the input is *u*_*i*_ and the output is *y*_*i*_. The set of hidden variables from the 0^th^ window, *g*_0_ are being fed to the next window. Thus, a recurrence is followed and the same unit with different parameter values is being repeated time and again till the final window. **(C)** Overall schematic representation of each *d*P-DCM recurrent unit consisting of neuronal, neurovascular, hemodynamic, and blood oxygenation level-dependent (BOLD) components. These components include respective latent variables, whose states are updated according to the associated equations (shown on the right, also refer to Equation 8). *x*_*E*2_*_i_* and *x*_*E*1_*_i_* refer to excitatory populations from regions 1 to 2, respectively. These regions are connected at the excitatory level. For each region, an inhibitory population exists, given by *x*_*I*1_*_i_*. Neuronal responses are the resultant of neuronal activity (due to the application of input stimulus) generated at the neuronal component. These excitatory responses induce vasoactive signals *s_*i*_*, which increase the blood flow *f*_*i*_. Changes in blood flow cause changes in blood volume (*v*_*i*_) and deoxyhemoglobin content (*q*_*i*_). Finally, these two hemodynamic states together yield BOLD response *y*_*i*_.

We have partitioned the time-series (including the inputs and the observed/measured BOLD fMRI responses) into *N* number of overlapping windows. Each such overlapping window *i* of duration *M* seconds moves from left to right as demonstrated in the Figure 1A. Please note that for illustration purposes the window size (*M*) is chosen to be 12 s in the Figure 1A. However, it can be easily adjusted and optimized for any given signal-to-noise ratio of the time series. The time course between 0 and 20 s has been magnified to show the overlap of two successive windows centered at 6th second and 7th second, respectively. Considering the total number of samples in a window being *T*, we have *M* = *T*Δ*t*. Thus, for every second, we have *T*/*M* = 1/Δ*t* samples (sampling frequency). The stride of these overlapping windows has been set to 1 s.

An unfolded version of the P-DCM-Recurrent Unit architecture through time is shown in the Figure 1B. For *i^th^* window, the input stimulus to the unit is *u*_*i*_ and the output fMRI BOLD response from the unit is *y*_*i*_. Each unit corresponds to one window and has four sub-units (components), namely, neuronal, neurovascular Coupling, hemodynamic and BOLD signal components (Figure 1C).

Every unit has two operations: (a) Forward Model and (b) Backward Model or Model Inference. In the Forward model, the model parameters and variables are initialized, and the fMRI BOLD response of the model is computed. To reduce the error between the calculated response and the observed fMRI BOLD response, we use model inference (as a part of the generative modeling technique) to update the parameters iteratively using gradient descent (Curry, 1944) until convergence is achieved (typically for 200 iterations or predefined tolerance threshold, usually 10^–6^). The unit has a similar structure to that of a vanilla Recurrent Neural Network (RNN) (Rumelhart et al., 1985), in which the same unit is being used again and again but with different set of inputs to get different sets of hidden variables and outputs. Please note that in the Forward modeling step, for the first window, we follow zero-mean initializations of the connectivity parameters as recommended by Friston et al. (2003), Havlicek et al. (2015). This has been done owing to the following reasons: (i) to ensure stability of the system (Friston et al., 2003), (ii) we compare task modulation with control condition, and therefore only the changes in baseline connectivity are of interest.

For any window *i*, we employ a *d*P-DCM-RU, which takes in input stimuli (for that window) and fits output BOLD responses (for the same window). In doing so, latent (e.g., neuronal, hemodynamic, vascular) responses are generated and parameters (e.g., connectivity) are inferred. Therefore, for any window *i* (*i* ∈ {0, 1, 2, …, *N–*1}), this set can be represented as: *g*_*i*_ = {Xi,Hi,WXi,WUi,WHi⁢WH⁢Xi⁢WB⁢O⁢L⁢Di}(see Figures 1B, C and Equation 8 below). The values of these parameters serve as the starting values or initializations for the next, i.e., (*i* + 1)*^th^* window. In other words, the parameters for window (*i* + 1) are initialized with the predicted values for its previous window *i*, preserving continuity between adjacent windows. It is to be noted that before performing model inversion, we do zero-padding with half-window length on each end of the data so that we can cover the entire extent of the actual signal. Therefore, the computed connectivity at every window is centered at that window.

The output of each *d*P-DCM recurrent unit is the fMRI BOLD response. A recurrence is being followed because the same unit with different parameter values repeatedly performs the same task or operation (on input sequences) till the final window.

Dynamic effective connectivity is estimated using overlapping windows. That is, partitioning the time-series (including the inputs and the observed/measured BOLD fMRI responses) into *N* number of overlapping windows such that ∀ *i* ∈ {0, 1, 2, …, *N–*1}, we get:


(8) $$d\,P-D\,C\,M_{i}=$$

{Xi(t+Δt)= WXiXi(t)+WUiUdi(t)Hi(t+Δt)= WHiθHi(Hi(t))+WH⁢XixEi(t)+ωHiyi(t+Δt)= WB⁢O⁢L⁢DiθB⁢O⁢L⁢Di([qi⁢(t+Δ⁢t)vi⁢(t+Δ⁢t)])+ ωB⁢O⁢L⁢Di

**FIGURE 2:**
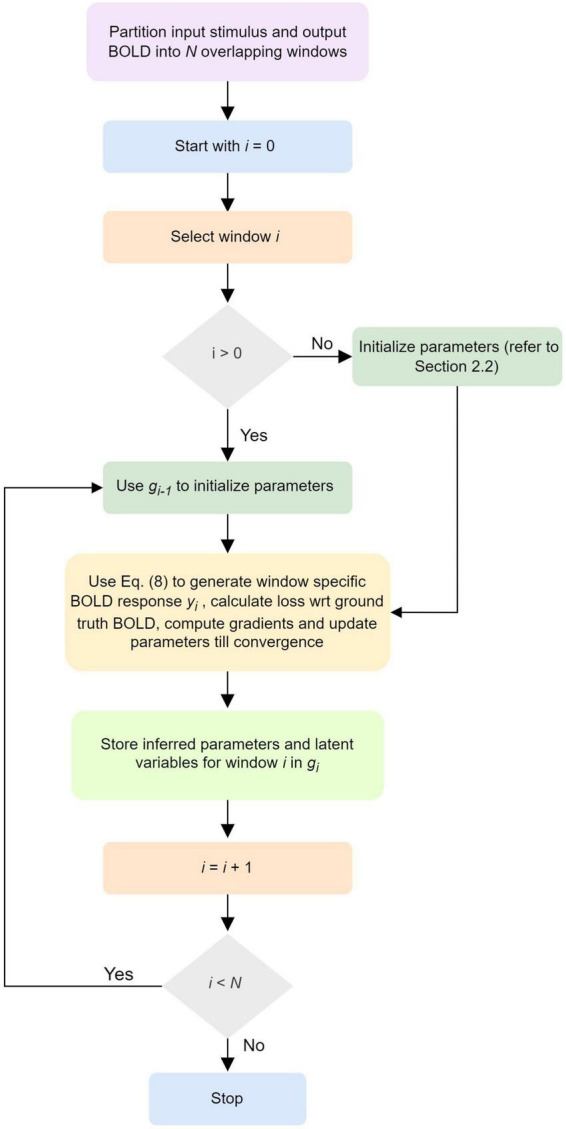
Workflow of *discretized* Physiologically informed Dynamic Causal Model (*d*P-DCM)-recurrent unit (RU).

A schematic illustrating the updates of the neuronal, neurovascular, hemodynamic, and BOLD variables (following the above Equation 8) in each *d*PDCM recurrent unit has been shown in the Figure 1C. Our proposed workflow has also been demonstrated in Figure 2 in a nutshell.

## 3. Simulations set-up and results

To check the face validity of the approach, first, we simulated connectivity profiles for 3- and 10-region graphical models (representing cognitive hypotheses), to show the ability of *d*P-DCM-RU to estimate dynamic effective connectivity and to distinguish different causal functional graphs using model evidence for a simple and a complex case, respectively.

### 3.1. Three-region model

#### 3.1.1. Case a: Time-varying connectivity

**FIGURE 3:**
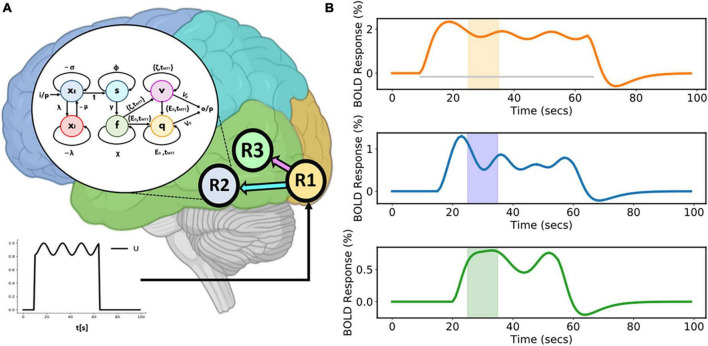
Three-region model and corresponding area-specific simulated functional magnetic resonance imaging (fMRI) blood oxygenation level-dependent (BOLD) responses. **(A)** Three-region model for which connections exist from Region 1 (R1) to Region 2 (R2) and from Region 1 (R1) to Region 3 (R3). The sinusoidal input *u* is applied to R1 and then activity propagates to both R2 and R3. The model inside the white circle (with black border) shows the internal sample model representation of R2. **(B)** Corresponding area-specific simulated fluctuating fMRI BOLD time courses with windows centered at the 30th second. The gray horizontal line represents the duration of the input stimulus.

This model comprises three regions (R1, R2, and R3) (as shown in Figure 3A). A sinusoidal input *u* is applied to R1 which then activates R2 and R3. Area-specific time-varying fMRI BOLD responses, given as a percentage signal change are shown in the Figure 3B. The colored boxes correspond to the recurrent window size for each of these time courses.

##### 3.1.1.1. Forward simulation

**FIGURE 4:**
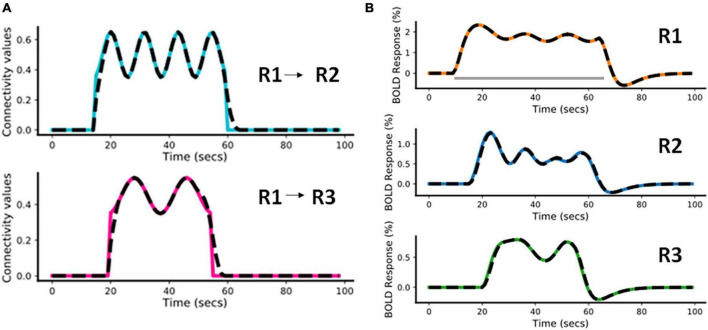
Predicted estimates along with the ground truth values for Case a. The black dashed lines represent the predicted responses and the colored time courses are the ground truth values. **(A)** Connectivity time courses, **(B)** area-specific functional magnetic resonance imaging (fMRI) blood oxygenation level-dependent (BOLD) time courses each expressed as a percentage change in response. The gray horizontal bar represents the stimulus duration.

In this example, we have considered a fast-varying connection from R1 to R2 and a slow-varying connection from R1 to R3. The assumed connectivity time courses between R1 and R2 and between R1 and R3 are shown in Figure 4A (colored plots). Supplementary Table 1A shows the piece-wise continuous functions used to simulate the connectivity values. The effective connectivity as a function of time (*t*) is denoted as *eff_conn*(*t*). Using the simulated connectivity pattern, we get the corresponding area-specific fMRI BOLD responses as shown in the Figure 4B (colored plots). The value of Δ*t* is 1/32s for all simulations (Wang et al., 2018). For all other parameters of the model, we please see Supplementary Table 1B (also please refer to Supplementary Table 1A of Havlicek et al., 2015).

##### 3.1.1.2. Model inversion

For this model inversion, we assume the same connectivity graph as was used for the forward simulation. In Figure 4, the black dashed lines represent the predicted estimates from the model on top of the colored ground truth (i.e., forward simulated) values. It can be noticed that for both connectivity time courses (Figure 4A) and fMRI BOLD responses (Figure 4B) the fitting is highly accurate, with slight inaccuracies in the effective connectivity estimates during the initial rise and during return to baseline, indicating that sharp increases or decreases are smoothed out in the BOLD signal and model inversion.

The Normalized Root Mean Squared Error [NRMSE (Shcherbakov et al., 2013)]^1^ value averaged over the two connections (R1 and R2, R1 and R3) is 2.14% and the NRMSE value averaged over the fMRI BOLD responses from the three regions (R1, R2, and R3) is 0.91%. We have repeated the above simulation set-up for a higher frequency input and have done corresponding model inversion, whose results have been provided in the Supplementary Section 1.1.1.

##### 3.1.1.3. Model comparison

**FIGURE 5:**
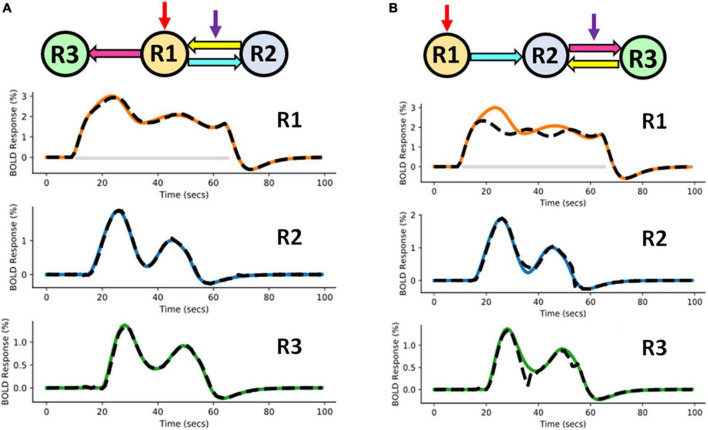
Competing hypotheses models **(A)** *m*_1_ (ground truth model) and **(B)** *m*_2_ (randomly chosen model) along with the corresponding area-specific functional magnetic resonance imaging (fMRI) blood oxygenation level-dependent (BOLD) responses. The black dashed lines represent the predicted responses and the colored time courses are the ground truth values. The red and purple arrows represent direct and modulatory inputs, respectively.

To show a noticeable difference in performance between two models during model comparison, we have considered an additional scenario. In this scenario, we have considered 2 competing hypotheses models *m*_1_ and *m*_2_ as shown in Figures 5A, B. In *m*_1_, *driving* input *u*_1_ (in red) is applied to R1, which is connected to both R2, and R3. Feedback connection exists from R2 to R1, which is influenced by *modulatory* input *u*_2_ (in purple). In *m*_2_, driving input *u*_1_ is applied to R1, which is connected to R2, which in turn is connected to R3. Feedback connection exists from R3 to R2. Connection from R2 to R3 is modulated by input *u*_2_. Hypothesis model *m*_1_ has been used for generating the ground truth BOLD data.

###### 3.1.1.3.1. Predictions using m_1_ and m_2_

In Figures 5A, B, the black dashed lines represent the predicted estimates from the model on top of the colored ground truth (simulated) values.

For *m*_1_, it can be noticed that the fitting of the BOLD responses is accurate for all three regions (Figure 5A). The NRMSE value for this reconstructed fMRI BOLD time-series with respect to the ground truth time-series is 1.45%. For *m*_2_, the errors of the predicted BOLD responses are higher compared to those of *m*_1_ (Figure 5B). This can be attributed to the absence of feedback connection from R2 to R1 in model *m*_2_. The NRMSE value^2^ thus has increased to 10.31%. Hence, in terms of accuracy, *m*_1_ performed better than *m*_2_, ΔNRMSE (= NRMSE_*m*2_–NRMSE_*m*1_ = 8.86%) is high. This demonstrates that our method is able to adequately distinguish between two cognitive hypotheses.

In addition to the above *m*_2_, we have the also evaluated performances of 10 more three-region models with randomly chosen configurations (Supplementary Section 1.1.2). ΔNRMSE values of these models with respect to *m*_1_ (Supplementary Figure 2) clearly show that *m*_1_ is superior in terms of performance and *m*_2_ is one of the less inferior models in the group (*m*_2_–*m*_12_).

### 3.2. 10-region model

**FIGURE 6:**
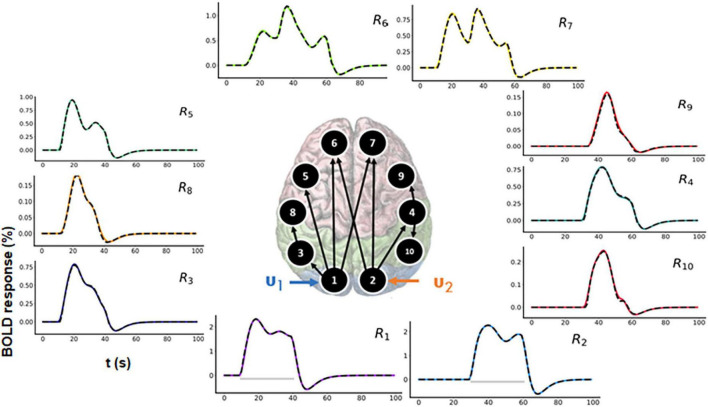
Functional magnetic resonance imaging (fMRI) blood oxygenation level-dependent (BOLD) time courses for 10-region model where connections exist between R1 to R3, R3 to R8, R1 to R5, R1 to R6, R1 to R7, R2 to R6, R2 to R4, R4 to R9, and R4 to R10. Two time-varying inputs *u*_1_ and *u*_2_ are applied to R1 and R2, respectively. The black dashed lines represent the predicted responses, and the colored lines represent the simulated time courses.

In a typical fMRI experiment, more than three brain areas are active. Thus, to demonstrate scalability, we evaluated a 10-region model as shown in the Figure 6 (center). The connectivity graph for the forward simulation is illustrated in the Supplementary Figure 4 (colored plots). Two time-varying inputs *u*_1_ and *u*_2_ (see Supplementary Figure 3) are applied to R1 and R2, respectively (see Figure 6 for the time courses). There is a delay of 20 s between these two inputs (see Supplementary Figure 3). Activity then propagates from R1 and R2 to the remaining regions.

#### 3.2.1. Forward simulation

The assumed connectivity time courses are shown in the Supplementary Figure 4 (colored plots). Using the model and the connectivity time courses, we get the corresponding area-specific fMRI BOLD responses.

#### 3.2.2. Model inversion

In both Figure 6 and Supplementary Figure 4, the black dashed lines represent the predicted estimates from the model on top of the colored ground truth (simulated) values. The prediction has a low NRMSE value (averaged over all the 10 regions) of 1.17%. The predicted connectivity time courses are also shown in Supplementary Figure 4. As can be seen, the predictions follow the ground truth time courses very closely for all brain areas.

#### 3.2.3. Model comparison

**FIGURE 7:**
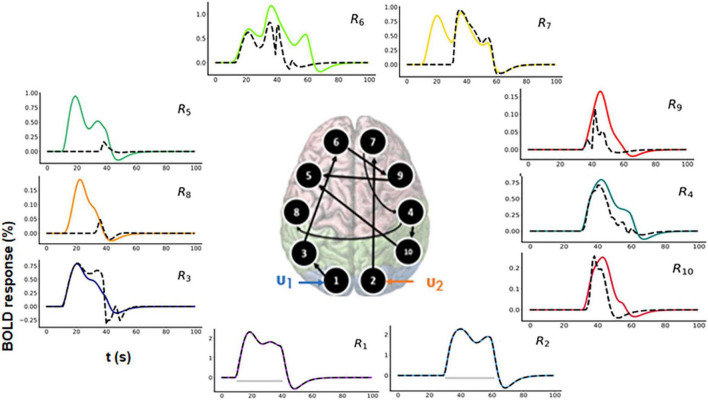
Predicted area-specific functional magnetic resonance imaging (fMRI) blood oxygenation level-dependent (BOLD) time courses along with the ground truth values for *m*_2_ which is a randomly chosen model. The black dashed lines represent the predicted responses and the colored time courses are the ground truth values. There are fitting inconsistencies for R3, R4, R5, R6, R7, R8, R9, and R10 fMRI BOLD time courses.

For model comparison purposes, we additionally performed model inversion for randomly selected model *m*_2_ as shown in Figure 7 (center).

##### 3.2.3.1. Prediction using *m*_2_

In Figure 7, the black dashed lines represent the predicted estimates from the model on top of the colored ground truth (simulated) values. Please notice that for fMRI BOLD responses the reconstruction and hence the fitting is good in the earlier brain areas, such as R1 and R2, but the discrepancies become more pronounced and errors larger for the later brain areas. The predicted connectivity time-courses are shown in Supplementary Figure 5. The NRMSE value (averaged over all the regions) for this reconstructed fMRI BOLD time-series with respect to the ground truth time-series is 25.75%. Hence, in terms of accuracy, *m*_1_ performed better than *m*_2_, ΔNRMSE (= NRMSE*_m_*_2_–NRMSE*_m_*_1_ = 24.58%) is high showing that our method can sufficiently differentiate between two cognitive hypotheses. Additionally, we have also considered another 10-region model with reciprocal connections and have performed corresponding model inversion with a randomly chosen configuration as shown in the Supplementary Section 3, confirming that *m*_1_ can be distinguished from a model with erroneous connectivity graphs.

## 4. fMRI BOLD time courses with measurement noise

In this case, we have used two models- a Three-region model (from “section 3.1.1 Case a: Time-varying connectivity”) as a simple model and a 10-region model (from “section 3.2.1 Forward simulation”) as a complex model. Here, the fMRI BOLD time courses are noisy with measurement or physical noise being present. Measurement or physical noise is an external noise which gets added to the signal while it is being acquired. For simulation purposes, this measurement noise used by us is a zero mean gaussian random noise with varying levels of standard deviation. Contrast-to-Noise Ratio (CNR) values were computed with respect to the ground truth values for fMRI BOLD responses. It is worth noting that we have defined CNR as the ratio between the standard deviation of the signal and the standard deviation of the noise (Definition four of CNR, Welvaert and Rosseel, 2013) and this definition is consistent with the previous DCM works (Friston et al., 2003; Frässle et al., 2017)^3^.

**FIGURE 8:**
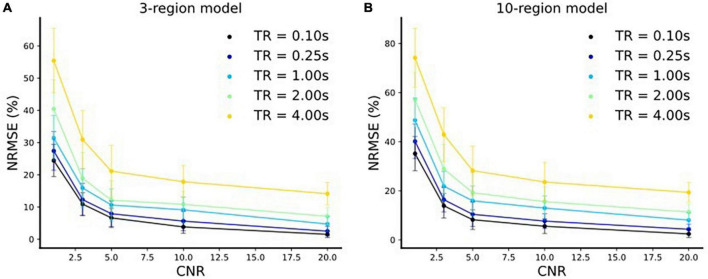
Normalized Root Mean Squared Error (NRMSE) (mean ± s.d.) expressed as a % vs. CNR for. **(A)** Three-region model and **(B)** 10-region model for five different repetition time (TR) settings.

The extracted fMRI time series stem from Principal Component Analysis (PCA) over numerous voxels in local volumes of interest which suppresses noise (Frässle et al., 2017). Therefore, as outlined in Frässle et al. (2017), the typical CNR levels (SNR in DCM terminology, see footnote 3 for further clarification) of fMRI time series used for DCM are three or more. It is to be noted that this value is highly dependent on the tasks and brain areas investigated. That is, for some tasks and/or brain areas, DCM and its variants and the derived connectivity values are unreliable. This is already true for DCM estimating only one connectivity profile for a run and even more so for estimation of time-varying connectivity. Please note that this is also true for estimation of time-varying functional connectivity using resting-state. That is, it is not our claim that our approach (and no other statistical approach) will work for any task and/or brain area or any MRI acquisition parameters (such as field strength, sequence, etc.) but only if certain conditions, such as CNR levels, are met that time-varying effective connectivity can be estimated using our approach. Therefore, we have conducted the simulations for 3- and 10-region models with different CNR values [CNR = (1, 3, 5, 10, 20)] and repetition times [TR = (4, 2, 1, 0.25, 0.1 s)]. In each case, we resampled (*via* linear interpolation) the timeseries to sampling frequency of 32 Hz resulting in an increase in the number of samples^4^. Using our method, we did model inversions for each of these settings for both three-region and 10-region models. We compared the estimated effective connectivity time courses to those of the simulated ground truth using Normalized Root Mean Squared Error (NRMSE), expressed as a percentage (%) as shown in Figure 8. We repeated the above setup for 10 runs with newly sampled noise and reported the mean and standard deviations (s.d.) of the NRMSE values over these 10 runs in Figure 8.

We make the following two major observations for both three-region (Figure 8A) and 10-region (Figure 8B) models:

(i) The respective NRMSE values decrease with increase in the CNR levels for each TR setting. Moreover, at any TR value we observe that the standard deviations decrease with an increase in the CNR level, as expected. This indicates that the reliability of predictions increases toward high CNR values (i.e., when the data is less noisy).

(ii) The corresponding NRMSE values decrease with a decrease in the TR value for each CNR level. Notably, at any CNR level, the standard deviations also typically decrease with a decrease in the TR value (i.e., when the sampling rate is high) indicating more reliable predictions.

## 5. Discussion

The fMRI signal is an indirect reflection of neuronal activity, mediated by neurovascular coupling and hemodynamics. Generative models describe the biophysical basis underlying fMRI and present a framework to interpret empirical observations. Through model inversion, generative models enable investigations of underlying neuronal dynamics and functional integration in the brain. One such state-of-the-art generative model is the Physiologically informed Dynamic Causal Modeling (P-DCM, Havlicek et al., 2015). Most existing DCM studies (Friston et al., 2003; Stephan et al., 2008; Moran et al., 2009; Havlicek et al., 2015) typically consider the effective connectivity to be static for a cognitive task within an experimental run. However, experimental conditions can vary with time, especially in cases of complex stimuli, e.g., movie, music, etc. Consequently, the connectivity strengths between disparate brain regions involved in processing these complex stimuli may fluctuate with time. Please note that in the conventional DCM framework (Friston et al., 2003; Stephan et al., 2008; Moran et al., 2009), it may also be possible to model dynamic connectivity by utilizing dynamic **B** or **C** matrices (please refer to Equations 2a–f for definition of **B** and **C**). However, such an approach requires prior knowledge of the time-varying connectivity profiles and just estimates the strength of these dynamic connectivity predictors. In contrast, our method does not require prior knowledge of connectivity profiles.

In the recent years, there has been an increasing number of studies to elucidate the dynamic (functional) connectivity in fMRI by investigating the temporal correlations of resting-state BOLD fluctuations in distributed brain areas (Cribben et al., 2012; Handwerker et al., 2012; Calhoun et al., 2014; Monti et al., 2014). In such Dynamic Functional Connectivity (DFC) studies, one of the predominant methods is to employ a sliding window-based approach to find the time-varying correlations (Chang and Glover, 2010; Kiviniemi et al., 2011; Jones et al., 2012). However, lacking a generative model, the correlations between the areas are determined on the level of observations but not on the level of the underlying causes (Stephan et al., 2010). In contrast, DCM accounts for the indirect nature of the BOLD signal and fits BOLD signals in the different ROIs using a system of differential equations (Friston et al., 2003; Stephan et al., 2008; Havlicek et al., 2015, Havlicek et al., 2017b).

In this paper, we have introduced *discretized* Physiologically Informed Dynamic Causal Model with Recurrent Units (*d*P-DCM-RU) to characterize dynamic effective connectivity of various brain regions during tasks. This method is a combination of two approaches, namely, a Euler based discretization technique and a recurrent sliding window approach for dynamically modeling fMRI BOLD responses and for exploring the causal interactions between different neuronal populations. To validate, we have carried out simulations with 3- and 10-region models. To that end, we have decomposed effective connectivity into static and dynamic components. The static component acts as a baseline component and the dynamic component varies with time sinusoidally. However, please note that our recurrent window-based parameter estimation method can predict any connectivity profiles.

For the Three-region model, we have considered two different connectivity graphs. Using the first example, we have simulated *noiseless* time-varying effective connectivity between the regions. The results show that the fits of fMRI BOLD responses and the effective connectivity have low error values, compared to the ground truth (i.e., forward simulated) BOLD responses. This is not a trivial result as even assuming the correct connectivity graphs does not guarantee model invertibility due to potential ill-posed problem.

To show a noticeable difference in performance between models during model comparison, we have selected another example scenario (Three-region model) with feedback from R2 to R1 modulated by an input (i.e., the ground truth model, *m*_1_) and 11 randomly chosen models for comparison. Results show that the randomly chosen models did not perform well in terms of fitting accuracy values (given by NRMSE) relative to the ground truth model (*m*_1_). The results indicate a clear distinction between the hypothesis models and demonstrates that the *d*P-DCM-RU approach does not necessarily guarantee a good fit to any BOLD signal and is therefore capable of distinguishing models.

Typically, more than three brain regions are active during a complex cognitive task. Therefore, to illustrate a more complex case, we have performed simulations for a 10-region model (also see 10-region model example with reciprocal connections in the Supplementary Section 3). Results clearly suggested that the method was able to predict effective connectivity with low error values and to fit fMRI BOLD responses with high accuracy. We did model a competing 10-region connectivity graph (hypothesis model) for comparison. Model inversion results demonstrated that this randomly chosen hypothesis model was unable to reconstruct fMRI BOLD signals accurately leading to large deviation values from the ground truth fMRI BOLD responses, in particular, in areas most distant from the input areas. In this 10-region example (“section 3.2 10-region model”), we did not provide any delay between the input and connectivity (e.g., R1 to other regions). However, the window/duration of connectivity and input do not necessarily have to match. That is, connectivity from a certain brain region to another brain region may change from baseline value later than the input depending on the experiment (for such an example, please refer to “section 3.1.1 Case a: Time-varying connectivity”).

In addition, we have considered noisy scenarios by adding measurement noise (see “section 4 fMRI bold time courses with measurement noise”). We have conducted simulations for 3- and 10-region models with different Contrast-to-Noise Ratio (CNR) values [CNR = (1, 3, 5, 10, 20)] and repetition times [TR = (4, 2, 1, 0.25, 0.1 s)]. We observed that the connectivity prediction error decreases and the reliability of predictions increases with an increase in CNR and a decrease in TR values, respectively (see Figure 8). Depending on the threshold for accuracy used, estimation of dynamic connectivity using our method may be prohibited (the same argument also applies for dynamic functional connectivity calculation). In the cases of low CNR and high TR values, standard denoising procedures may be used, which may lead to less erroneous and more reliable parameter estimates.

One of the foremost limitations of DCM in general is that they are restricted to a fixed number of regions because of their computational demand (Frässle et al., 2017). In particular, the number of coupling parameters (i.e., connectivities) grows quadratically with the number of nodes, and therefore the computational time required to invert these models grows exponentially with the number of free parameters (Seghier and Friston, 2013; Frässle et al., 2017). Since our method is based on the P-DCM framework, the above problem also persists in our case. Additionally, the windowing scheme makes the approach even more computationally intensive. Furthermore, higher-order integrators are potentially slow in practice and computational (memory) requirements become even larger because of explicit Jacobian-based update schemes, which are evaluated numerically at each time point (Friston et al., 2003). Therefore, to increase computational speed and reduce memory, we utilize the lower-order Euler method. However, a disadvantage of the Euler method compared to higher order ODE solvers is lower numerical accuracy. Nevertheless, the numerical errors can be kept low for small step size Δ*t* values. Following Wang et al. (2018), we have assessed the impact of different step sizes for the three-region model. As illustrated in the Supplementary Figure 10, NRMSE is the lowest (with a value of 0.82%) for Δ*t* = 1/32 s and it increases (more than linearly) with step-size (e.g., for Δ*t* = 1/4 s, NRMSE = 19.97%). Hence, we selected Δ*t* = 1/32 s for all our simulations.

Another challenge for *d*P-DCM-RU is the selection of optimal window size. If the window sizes are too large, then the transients may not be captured, whereas too small window sizes may lead to overfitting of the model (Sakoğlu et al., 2010; Shakil et al., 2016). We investigated the effect of window sizes on the (three-region) model performance given in terms of NRMSE (%). Supplementary Figure 11 shows that for a window size of 5 s, NRMSE is low (1.45%), whereas model performance substantially degrades for a window size of 11 s (NRMSE increases to 22.68%). However, the window size can be easily adjusted to the complexity of the experimental design. A 5 s window size worked well for varying connectivity chosen in our simulations (Supplementary Figure 11). Please note that—as the BOLD signal can be represented as a smoothing kernel—very fast neuronal dynamics cannot be recovered by fMRI for any window size, independent of acquisition speed and analysis method (Polimeni and Lewis, 2021).

Park et al. (2018), Zarghami and Friston (2020) have proposed dynamic functional connectivity estimation frameworks for resting state fMRI (rs-fMRI). Park et al. (2018) utilized discrete cosine transform eigenvariates and Hierarchical Parametric Empirical Bayes (PEB) approach (Friston et al., 2016) to model dynamic functional connectivity at two levels. In the first level, they have inverted a spectral DCM (spDCM) separately for each window to obtain (within-window) connectivity parameters. Subsequently, in the second level they have applied PEB to estimate between-window effects on these connectivity parameters. Motivated by the nonlinear dynamical systems theory, Zarghami and Friston (2020) proposed a hybrid generative framework consisting of Hidden Markov Model (HMM), PEB and spDCM (discrete and continuous hierarchical models) to explain metastable dynamics in the brain via modeling the temporal evolution of different connectivity states. Their paradigm utilized the variational message passing technique, for which the HMM provided Bayesian model averages for the intermediate PEB level, which successively supplied priors to each spDCM. In this manner, by assigning an itinerant prior to the state-transition matrix, they estimated the transition and state-dependent effective connectivity parameters. On the contrary, our approach (“section 2.2 Time varying calculations of the model parameters”) neither requires “two-level” connectivity estimation nor involves a “hybrid” generative approach. Furthermore, these previous studies have typically considered larger window sizes for their estimations, which they have shown to work well for rs-fMRI. It is important to note that in contrast to our approach, these works do not capture the entire temporal extent of connectivity dynamics (i.e., connectivity values do not exist for all time-points) and are only suitable for tracking slow dynamics (Park et al., 2018, Zarghami and Friston, 2020). Furthermore, our method considers the stride of the overlapping windows to be 1 s and, therefore, covers the entire extent of the signal (in the order of seconds), and is able to track relatively faster dynamics (as demonstrated in Figure 4, where we have considered slow and fast varying connectivities). Finally, our work deals with task-based fMRI and not rs-fMRI.

Another recent work (Wang et al., 2018) utilizing Recurrent Neural Networks (RNNs) (Rumelhart et al., 1985; Hochreiter and Schmidhuber, 1997) proposed a biophysically interpretable DCM-RNN. Although both *d*P-DCM-RU and DCM-RNN take inspiration from the recurrence concept as in RNNs, DCM-RNN differs from *d*P-DCM-RU in many aspects: In their method, they have used Truncated Backpropagation Through Time (TBPTT) for computing parameter updates, whereas we have simply used gradient descent as done in standard DCM implementations (Havlicek et al., 2015). Their definition of recurrence is the same as in standard RNNs utilizing segmented batches. Therefore, they have used multiple batches in *parallel* (as in deep learning) and updated model parameters *via* TBPTT. To ensure that the gradients obtained by TBPTT are reliable, each of these segments has to be sufficiently long and the sampling time has to be sufficiently small. Since they use parallel batches for parameter update, the gradients may often not be accurate (Wang et al., 2018). This is because each of those batches does not represent the characteristic of the whole signal. Due to this batch *parallel* processing, they can only estimate *static* connectivity. In our case, the recurrence lies between the successive P-DCM units. We do *sequential* processing of each such unit estimating time-varying connectivity parameters. Furthermore, unlike DCM-RNN, we have used the state-of-the-art DCM model, i.e., P-DCM (Havlicek et al., 2015) in our framework instead of Single-State DCM (S-DCM) (Friston et al., 2003). Finally, the authors of DCM-RNN claimed that their model can be extended for complex paradigms such as movie watching using representations of the complex stimuli. However, at this stage their model cannot estimate dynamic connectivity without further modifications.

One of the major DCM steps is to conduct a Model Selection (using group Bayes factor) between several alternative competing models to establish which model accounts best for the experimental observations. After selection of the optimal model, making further inferences about its parameter estimates (e.g., connectivity) is typically not done in DCM studies and usually some statistical values (e.g., mean) of the parameter estimates for the group is reported (Stephan et al., 2009). However, inferences about model parameters can still be made with either a fixed effects or a random effects approach. For fixed effects parameter inference, a common way is to use Bayesian average (for example “DCM average” function in SPM) (Friston et al., 1994). For random effects parameter inference, subject-specific parameter estimates can be used with a classical frequentist test, such as a paired *t*-test (between model parameters) or repeated measures ANOVA in case of multiple sessions per subject (Stephan et al., 2009).

It is important to note that our proposed method is not necessarily restricted to block designs. Naturalistic stimulus typically comprises block (e.g., fluctuation of light intensity) and event-related (e.g., presence of face for a limited period of time) components. However, that due to sluggishness of the hemodynamic response, very fast events may not be detected, similarly as in standard fMRI data and analysis. Please note that our approach can be used with arbitrary time-varying inputs and the choice of sinusoidal inputs (in our simulations) is for illustrative purposes and can easily be modified.

In our approach, the estimates from the previous window serve as the initial values (and not constraints) for the next window. Therefore, the model gets a good starting point which makes it easier to optimize. However, for any window, model inversion is performed independently, therefore, an estimation error in the previous window will unlikely be reflected in the next window. Furthermore, for optimization we have resorted to using the conventional gradient based scheme, i.e., gradient descent. Although gradient descent can potentially be slow, it has a better generalization performance (Zhou et al., 2020). Alternatively, one can use momentum based (Sutskever et al., 2013) or adaptive algorithms (Kingma and Ba, 2015) to further speed up performance while maintaining accuracy (Ruder, 2016). Nonetheless, we have not explored different optimization algorithms and strongly feel that it is beyond the scope of the current paper since this is a general topic for all DCM and model inversion approaches and not specific to our paper. To summarize, when a subject is exposed to a complex stimulus (e.g., watching a movie), human brains show dynamic effective connectivity between remote areas on the neuronal level, which can be indirectly measured using fMRI and which can be effectively recovered using the *d-*PDCM-RU approach. In the future, we will demonstrate the validity of our method in clinical and cognitive neuroscience studies.

## Data availability statement

The original contributions presented in this study are included in the article/Supplementary material, further inquiries can be directed to the corresponding authors.

## Author contributions

SN and KU made substantial contributions to the conceptualization, methodology, design of the experiments, data analysis, visualization, and drafting the manuscript. Both authors provided final approval of the submitted version of the manuscript.

## Footnotes

1 NRMSE is defined as the ratio of Root Mean Squared Error (RMSE) to the difference between the maximum and minimum values of the ground truth data (Shcherbakov et al., 2013).

2 In noiseless cases, as in some of the current simulations, Free Energy (Friston et al., 2003) formulation cannot be utilized. Instead, NRMSE values between the fits (with respect to ground truth) obtained using different models can be used to indicate, which model is more accurate than the other. NRMSE is independent of the signal strength or amplitude and therefore, a more robust indicator of accuracy (while comparing different models) than RMSE.

3 There exist several definitions of SNR and CNR: SNR is typically defined as the ratio of the mean of the fMRI signal to the standard deviation during baseline. However, the one that is predominantly used in the DCM literature is given as the ratio between the standard deviation of the signal and the standard deviation of the noise. This definition is the same as the Definition 4 of CNR in Welvaert and Rosseel, 2013. Therefore, following Welvaert and Rosseel, 2013, we will be using CNR terminology in our paper.

4 We have used linear interpolation technique for upsampling/resampling. Other interpolation/resampling methods (cubic, spline, etc.) and resampling frequencies have not been explored and it is beyond the scope of the current paper.
