# Supplementary material for: Dynamic Effective Connectivity using Physiologically informed Dynamic Causal Model with Recurrent Units: A functional Magnetic Resonance Imaging simulation study
Source: Front Hum Neurosci. 2023 Mar 1;17:1001848. doi: 10.3389/fnhum.2023.1001848 (PMC10014816; doi:10.3389/fnhum.2023.1001848)
Supplement: Supplementary file 1 [file Data_Sheet_1.pdf]

Supplementary Material

1 3-region model

1.1 Case a: Time-varying connectivity

Simulated Connection	Function
R1 → R2	$eff\_conn(t) = \{0.5 + 0.15 sin\left(\frac{2\pi}{11.25}(t-15)\right), for\ 15 \leq t \leq 60,$<br>$0, \quad otherwise$
R1 → R3	$eff\_conn(t) = \{0.45 + 0.1 sin\left(\frac{2\pi}{17.5}(t-20)\right), for\ 20 \leq t \leq 55,$<br>$0, \quad otherwise$

Table 1A. Corresponding piecewise continuous functions for simulated effective connectivity time courses.

Parameter	Name	Value
σ (Hz)	Excitatory self-connection	0.8 (0.1–1.5)
μ (Hz)	Inhibitory–excitatory connection	0.8 (0–1.5)
λ (Hz)	Inhibitory gain factor	0.1 (0–0.3)
φ (Hz)	Decay of vasoactive signal	0.6
ϕ (Hz)	Gain of vasoactive signal	1.5
χ (Hz)	Decay of blood inflow signal	0.6
α	Grubb's exponent	0.32
E_0	Oxygen extraction fraction at rest	0.4

Table 1B. Parameter values and their plausible ranges (in brackets) adapted from Table 1A of Havlicek et al., 2015.

1.1.1 Time varying connectivity with higher frequency input

1.1.1.1 Forward Simulation

The same simulation setup of Section 3.1.1 has been used here with a higher frequency input (Supplementary Fig. 1C). For further details about sampling time and model parameters, please see Section 3.1.1 in the main text.

1.1.1.2 Model Inversion

We have used *d*P-DCM-RU for model inversion to estimate the effective connectivity time-courses. In Supplementary Fig. 1, the black dashed lines represent the predicted estimates from the model on top of the colored ground truth (i.e., forward simulated) values. The NRMSE value averaged over the 2 connections (R1 and R2, R1 and R3) is 2.17 % and the NRMSE value averaged over the fMRI BOLD responses from the 3 regions (R1, R2 and R3) is 0.92 %. These results suggest that our model is able to infer connectivity parameters even with higher frequency inputs.

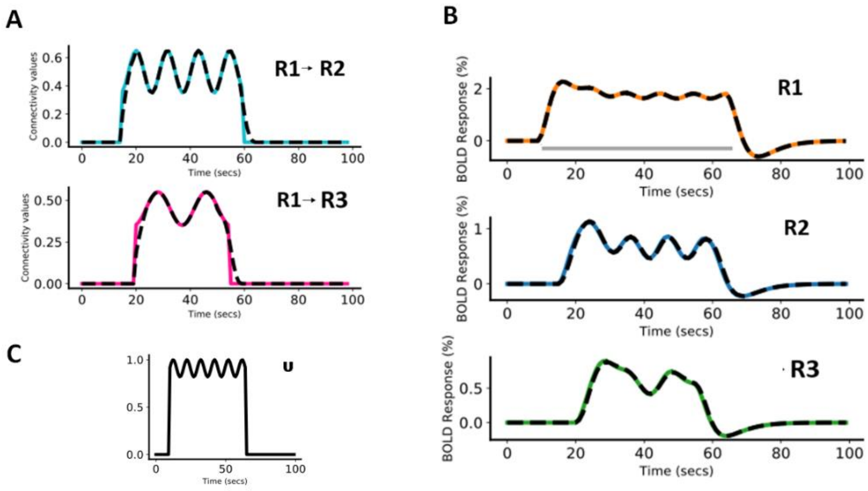

Supplementary Figure 1. Predicted estimates along with the ground truth values. The black dashed lines represent the predicted responses and the coloured time courses are the ground truth values (A) Connectivity time courses (B) Area-specific fMRI BOLD time courses each expressed as a percentage change in response (C) Higher frequency input used in the simulation.

1.1.2 Model Comparison

In addition to m_2, we have considered 10 more models with randomly chosen configurations, m_i, where $i \in \{3, 4, 5, \ldots, 12\}$. For all these randomly chosen models ($m_2 - m_{12}$), we have reported the Normalized Root Mean Squared Error differences (ΔNRMSE) with respect to m_1 (see Supplementary Fig. 2) and the values suggest that m_1 is superior to all the randomly chosen models in terms of accuracy. Moreover, the NRMSE for m_2 (highlighted in the bottom-right bar plot of Supplementary Fig. 2) depicts that m_2 is one of the less inferior models in the group.

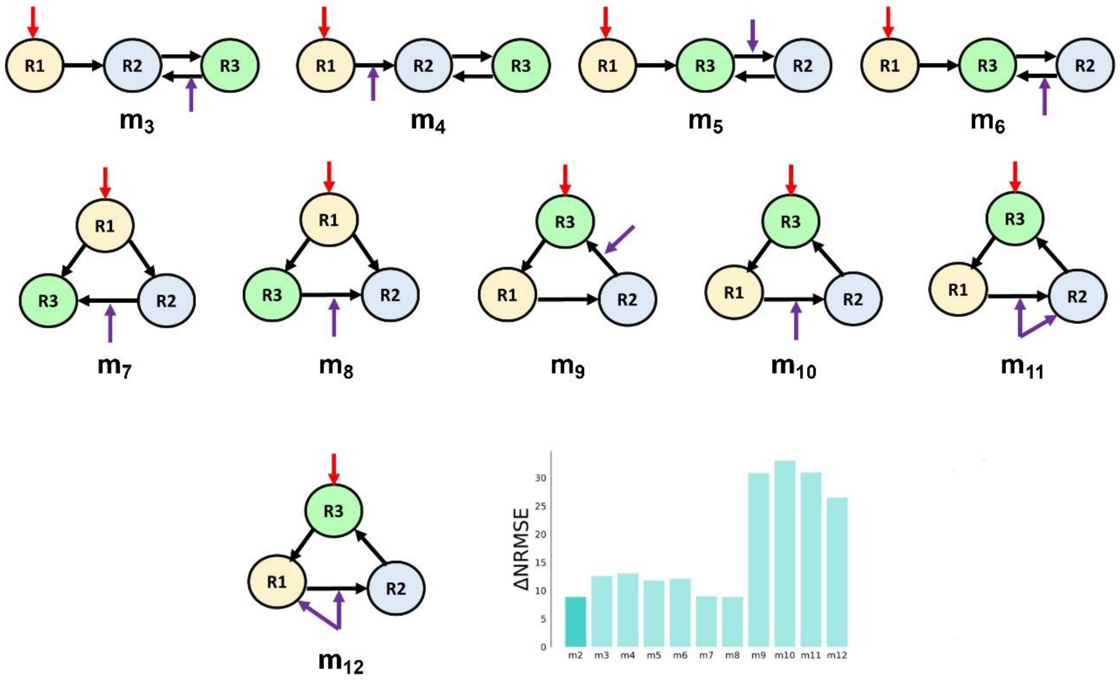

Supplementary Figure 2. Model Comparison Results for the 3-region model with randomly chosen configurations. Bottom-right bar plot indicates that (in terms of accuracy) m_1 is better than all the remaining models in the group and m_2 is one of the less inferior models.

2 10-region model

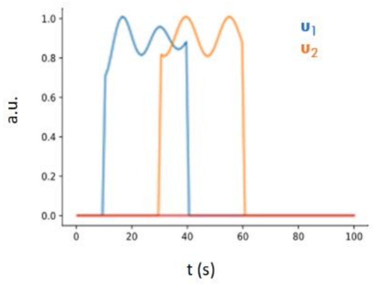

Supplementary Figure 3. Two time-varying inputs u_1 and u_2 applied to R1 and R2 respectively in the 10-region model.

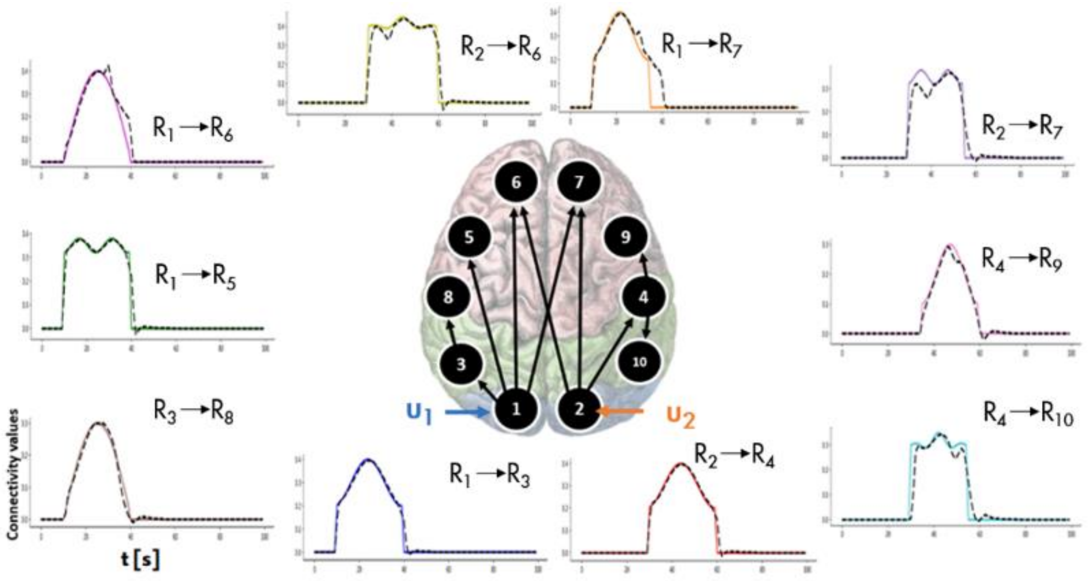

Supplementary Figure 4. Predicted effective connectivity time-courses (dashed) on top of ground-truth (simulated) effective connectivity time-courses for m_1 (true model) in the 10-region model.

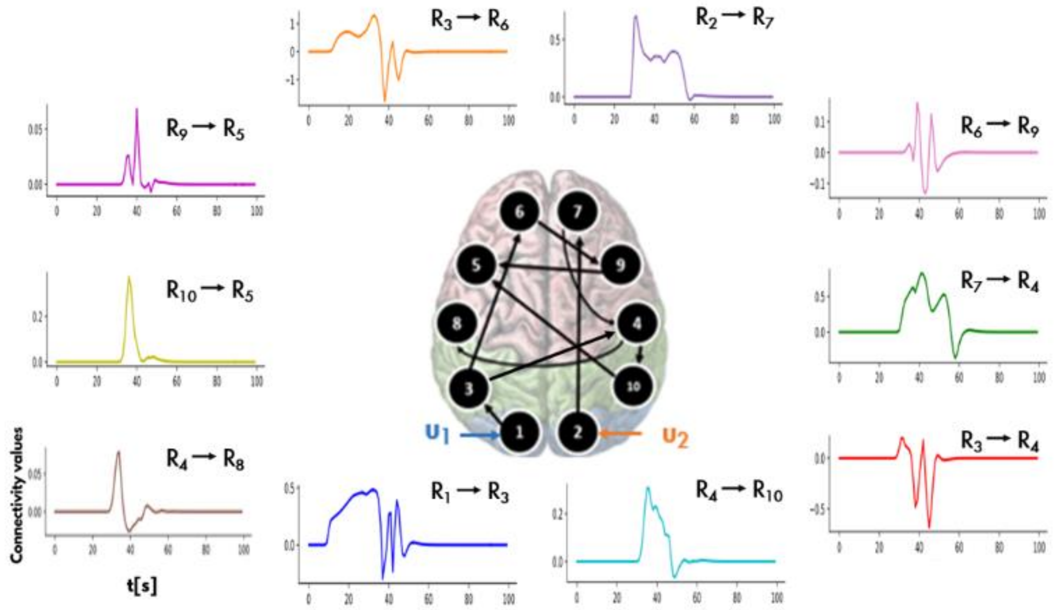

Supplementary Figure 5. Predicted effective connectivity time-courses for m_2 (randomly chosen model) in the 10-region model.

3 10-region model with reciprocal connections

In this example, we have considered a 10-region model (m_1) with reciprocal connections. The connectivity graphs for the forward simulation are illustrated in Supplementary Fig. 6A (center). Two time-varying inputs u_1 and u_2 are applied to R1 and R2, respectively (Supplementary Fig. 6B). The corresponding area-specific simulated fMRI BOLD responses are shown in Supplementary Fig. 6A (coloured plots).

In Supplementary Fig. 6A, the black dashed lines represent the predicted estimates from the model on top of the colored ground truth (simulated) values. The prediction has a low NRMSE value (averaged over all the 10 regions) of 1.64%. As can be seen, the predictions follow the ground truth time courses very closely for all brain areas. The predicted connectivity time courses (black-dashed lines) along with the simulated ones (colored) are shown in Supplementary Fig. 7.

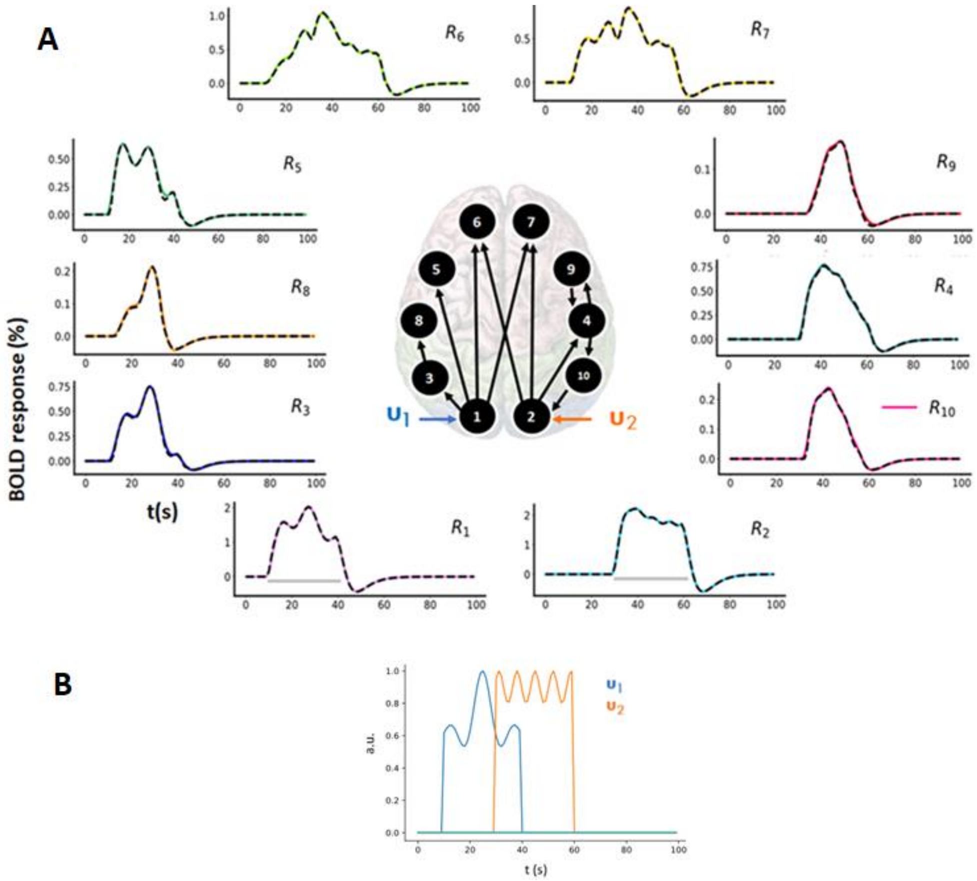

Supplementary Figure 6. (A) fMRI BOLD time courses for 10-region model with reciprocal connections. Two time-varying inputs u_1 and u_2 are applied to R1 and R2, respectively. The black dashed lines represent the predicted responses, and the colored lines represent the simulated time courses. (B) Two time-varying inputs u_1 and u_2 applied to R1 and R2 respectively.

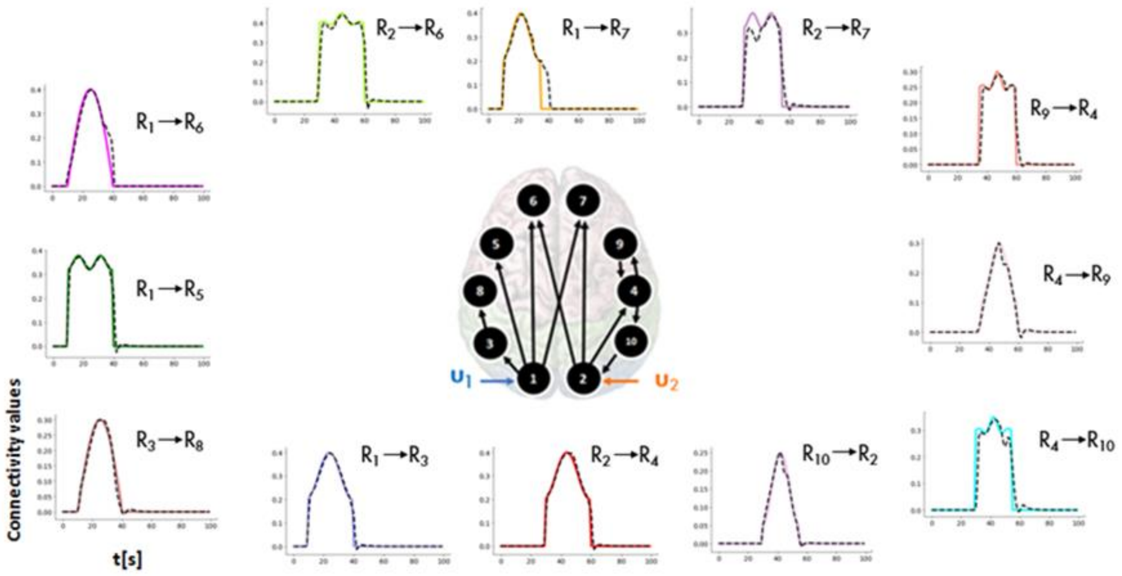

Supplementary Figure 7. Predicted effective connectivity time-courses (dashed) on top of ground-truth (simulated) effective connectivity time-courses for m_1 (true model) in the 10-region model with reciprocal connections.

3.1.1 Model Comparison

For model comparison purposes, we additionally performed model inversion for randomly selected model m_2 as shown in Supplementary Fig. 8 (center). Two time-varying inputs u_1 and u_2 are applied to R1 and R2, respectively (Supplementary Fig. 6B).

3.1.1.1 Prediction using m_2

In Supplementary Fig. 8, the black dashed lines represent the predicted BOLD estimates from the model on top of the colored ground truth (simulated) values. The fitting is good in the earlier brain areas, such as R1 and R2, but the errors become larger for the later brain areas. Predicted connectivity estimates are shown in Supplementary Fig. 9. The NRMSE value (averaged over all the regions) for this reconstructed fMRI BOLD time-series with respect to the ground truth time-series is 28.79%. Hence, in terms of accuracy, m_1 performed better than m_2, ΔNRMSE (= $NRMSE_{m2}$ – $NRMSE_{m1}$ = 27.15%) is high. This example illustrates that our approach works with complex models having reciprocal connections.

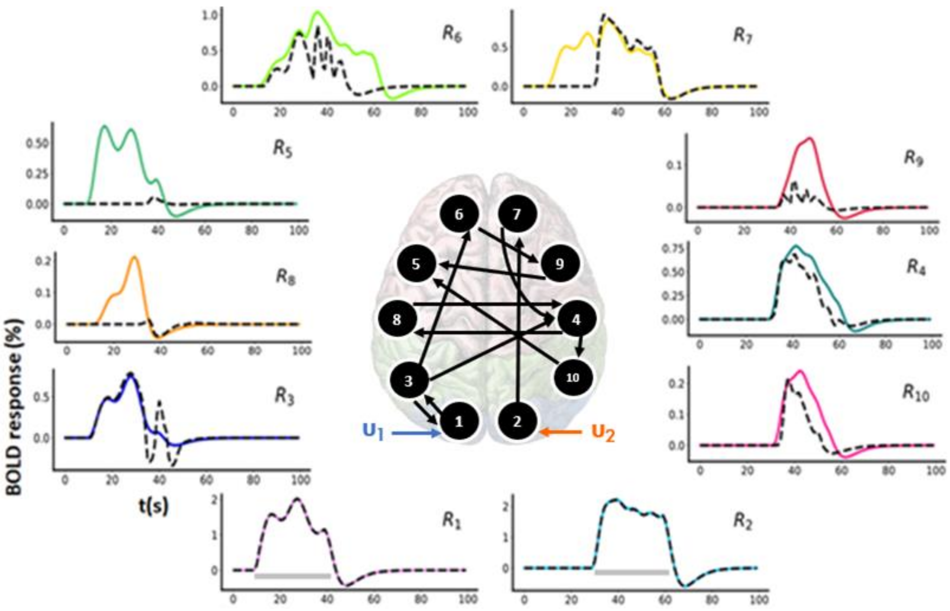

Supplementary Figure 8. Predicted area-specific fMRI BOLD time courses along with the ground truth values for m_2. The black dashed lines represent the predicted responses and the colored time courses are the ground truth values. There are large fitting errors for R3, R4, R5, R6, R7, R8, R9 and R10 fMRI BOLD time courses.

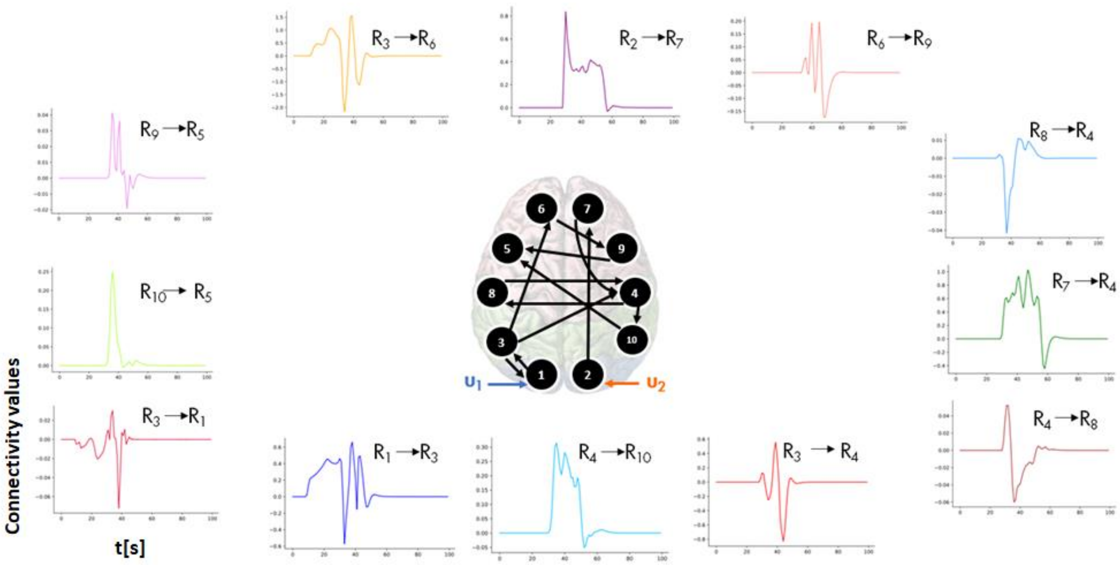

Supplementary Figure 9. Predicted effective connectivity time-courses for m_2 (randomly chosen model) in the 10-region model with reciprocal connections.

4 Ablations

4.1 Effect of step-sizes

In the Euler method, it is important to choose a small step size to reduce numerical inaccuracies. Following Wang et al., 2018, we study the effect of step-sizes on the model performance, as shown below in Supplementary Fig. 10. Using our 3-region model (Example 1, Section 3.1.1) we simulate

fMRI signals (realistic) with fifty different stable parameter configurations. In each of these configurations, fMRI signals generated with Δt=1/64s serve as the ground truth and others with Δt= {1/32, 1/16, 1/8, 1/4}s are compared against the ground truth (Wang et al., 2018). Please note that prior to comparison the ground truth signal is down sampled to the lower temporal resolution (Wang et al., 2018). We notice that with increasing step-sizes, the NRMSE (%) error grows more than linearly. Based on this observation, we selected Δt=1/32s as a suitable value for our simulations.

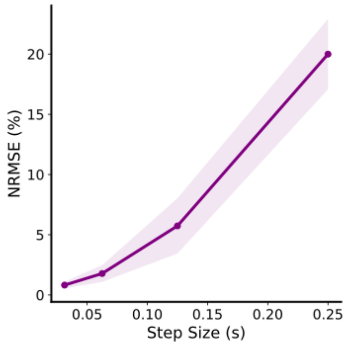

Supplementary Figure 10. Impact of various step-sizes on final model performance in terms of NRMSE values.

4.2 Effect of window sizes

Here, we assess the impact of window sizes and notice that the performance of the model (in terms of NRMSE %) substantially worsens with an increase in the size of the windows suggesting that longer windows are unable to capture the signal dynamics (Supplementary Fig. 11). Based on this observation we have selected a window size of 5 seconds in our simulations.

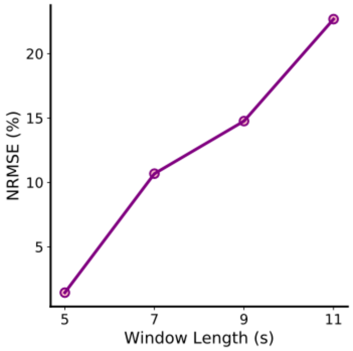

Supplementary Figure 11. Effect of window sizes on model performance.

References

Havlicek, M., Roebroeck, A., Friston, K., Gardumi, A., Ivanov, D., & Uludag, K. (2015). Physiologically informed dynamic causal modeling of fMRI data. Neuroimage, 122, 355-372.

Wang, Y., Wang, Y., & Lui, Y. W. (2018). Generalized recurrent neural network accommodating dynamic causal modeling for functional MRI analysis. NeuroImage, 178, 385-402.
